# Root coverage using a microsurfaced acellular dermal matrix: A retrospective case series

**DOI:** 10.1002/cap.10361

**Published:** 2025-07-28

**Authors:** Yu‐Chang Wu, Guo‐Liang Cheng, Shaun Rotenberg

**Affiliations:** ^1^ Division of Periodontology College of Dentistry, The Ohio State University Columbus Ohio USA; ^2^ Private Practice Columbus Ohio USA

**Keywords:** acellular dermis, dental esthetics, gingival recession, plastic surgery, wound healing

## Abstract

**Background:**

Acellular dermal matrices (ADMs) have been used for root coverage for over 25 years, yet few advancements have improved clinical outcomes or reduced complications. This case series evaluated the use of a novel microsurfaced ADM (mADM), which features a microtextured surface designed intended to promote healing and improve graft integration, for treating gingival recession defects.

**Methods:**

Eleven RT1 gingival recession defects from five patients were treated using mADM between January and May 2023 by a single surgeon (S.R.). A modified vestibular incision subperiosteal tunnel access technique was used for multiple recession defects, while a subperiosteal pouch technique was performed for single‐tooth recession defects. Clinical outcomes were assessed at baseline and 12 months. Pair *t*‐tests were utilized to compare changes overtime.

**Results:**

The mean recession depth reduced from 3.64 ± 0.50 mm to 0.73 ± 0.79 mm after 12 months. Keratinized tissue width increased from 2.32 ± 0.81 mm to 3.36 ± 0.92 mm. Gingival phenotype remained thick for all the cases. Significant root coverage was achieved (*p* < 0.05) with no graft exposure; complete root coverage was observed in 45.5% (5/11). Patients reported minimal discomfort and satisfactory healing.

**Conclusions:**

Within the limits of this retrospective case series, mADM may be considered a viable option for the treatment of RT1 gingival recession defects. Future randomized clinical trials should be performed to compare this matrix with other options to deal with recession defects.

**Key points:**

- In this case study, the novel microsurfaced acellular dermal matrices (mADM) demonstrated significant root coverage improvements in RT1 gingival recession defects, with a mean recession reduction from 3.64 to 0.73 mm at 12 months, achieving 80% root coverage and complete coverage in 45.5% of treated sites.
- The mADM may serve as a promising alternative to autogenous grafts, but larger‐scale randomized clinical trials are necessary to confirm long‐term efficacy and patient‐reported outcomes.

**Plain language summary:**

For decades, gum recession—when the gum tissue pulls back from the teeth and exposes the roots—has been treated with acellular dermal matrices (ADMs) made from donated cadaver tissue, avoiding the need to harvest tissue from the patient. Although traditional ADM is widely used, concerns exist regarding its healing and long‐term stability. In this case series, a new type of ADM called microsurfaced ADM (mADM) was used. Like ADM, mADM is derived from cadaver tissue but has a specially textured surface intended to support graft integration with the gums. Five non‐smoking patients were treated with mADM and followed for 12 months. The treatment resulted in excellent root coverage without complications such as graft exposure or infection. Patients reported very little discomfort and were satisfied with the esthetic outcome. Within the limits of this study, these findings indicate that mADM may offer a viable alternative for treating RT1 gum recession defects. Nonetheless, further randomized clinical trials are needed to compare its initial healing, long‐term outcomes, and patient‐reported outcomes with other treatment options.

## INTRODUCTION

Advancements in periodontal plastic surgery techniques and materials have continuously progressed to improve clinical outcomes. While the subepithelial connective tissue graft (SCTG) remains the benchmark for root coverage and augmentation of keratinized tissue,^1^ its use is limited due to the morbidity associated with a secondary surgical site.^2^, ^3^ Additionally, the anatomy of the hard palate can constrain the treatment of multiple teeth, necessitating multiple surgeries.^4^ For over 25 years, soft tissue augmentation involving the use of an acellular dermal matrix (ADM) has been utilized as an alternative to autogenous tissue. ADM eliminates the need for a donor site, enabling clinicians to address multiple sites in one procedure.^5^ However, studies indicate that ADM may offer less long‐term stability and minimal gains in keratinized tissue compared to SCTG.^5^, ^6^ Nonetheless, ADM remains a viable alternative to SCTG.^7^

ADM, sourced from human tissue and processed to remove dermal cells, retains a bioactive matrix.^8^ Initially developed for treating burn wounds in medicine,^8^ ADM has been integrated into dentistry to facilitate root coverage and to augment tissue thickness.^9^, ^10^ Despite widespread adoption, there have been limited advancements in ADM since its inception, primarily centered on optimizing storage and hydration methods.^11^

**FIGURE 1:**
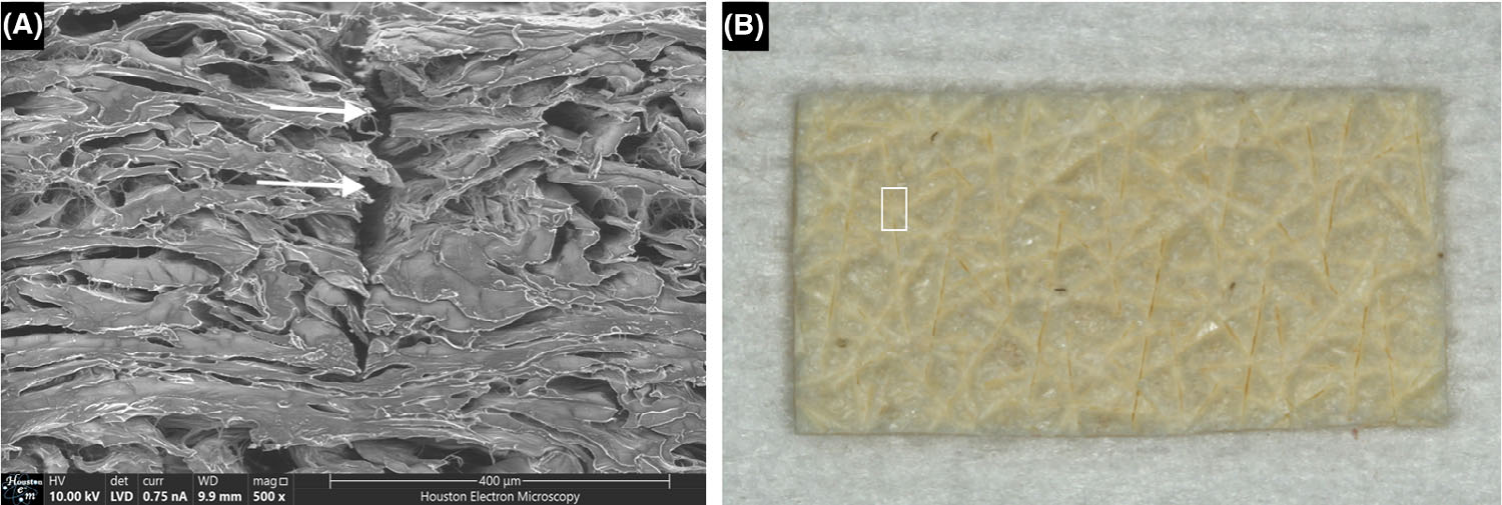
(A) Scanning electron microscopy (SEM) image of microsurfaced acellular dermal matrix (mADM) at 500× magnification, revealing a textured microcut surface that enhances surface area. The layered microstructure is irregularly arranged, with a visible central separation or cleft (indicated by arrows), further highlighting the microstructural variations. Scale bar = 200 µm. (B) Clinical image of a dehydrated 1 cm × 2 cm piece of mADM, demonstrating its micro‐textured surface. The rectangular box highlights a clinically visible microcut. Image provided courtesy of Osteogenics Biomedical. Copyright © 2025, Osteogenics, Lubbock, TX, USA. Used with permission.

A novel microsurfaced ADM^12^ (mADM; Microderm, Osteogenics Biomedical, Lubbock, TX, USA) was recently developed intended to enhance healing and clinical outcomes. mADM is a freeze‐dried allograft processed without the use of antibiotics that is microtextured to increase its surface area.^12^ The microsurfacing technique involves creating microcuts on the surface of ADM^12^ that are visible clinically and in more detail with the use of scanning electron microscopy (Houston Electron Microscopy) (Figure 1). Marinelli et al.^13^ conducted a study comparing mADM to conventional ADM in treating 20 patients with deep burn wounds. The findings indicated that mADM led to increased cell infiltration, improved graft integration, and reduced graft shrinkage between 12 and 19 days postoperatively compared to traditional ADM.^13^

The purpose of this report is to describe the usage of mADM in the treatment of gingival recession for root coverage in five cases with descriptions and outcomes up to 12 months.

## MATERIALS AND METHODS

### Study design and population

This retrospective case series included five non‐smoking patients (aged 38‒62 years), presenting with a total of 11 teeth requiring treatment, sought care primarily due to concerns about periodontal health and tooth sensitivity associated with gingival recession (RT1)^14^, ^15^ caused by aggressive tooth brushing. These were the first five mADM‐treated cases at a periodontal specialty practice in Columbus, Ohio (January‒May 2023), each with at least 1 year of follow‐up.

All surgeries and clinical measurements (Tables 1 and 2) were performed by a single experienced periodontist (S.R.) in a private practice setting. None of the surgical sites had undergone any previous surgical intervention. Baseline clinical measurements (Table 1), including recession depth (REC), clinical attachment level (CAL), gingival phenotype, and keratinized tissue width (KTW), were recorded on the mid‐buccal aspect of the tooth, and recorded to nearest mm using a UNC‐15 periodontal probe both preoperatively and 12 months postoperatively. The gingival phenotype was assessed based on the transparency of the periodontal probe when inserted into the gingival sulcus.^16^ The presence/absence of the cementoenamel junction (CEJ) and cervical discrepancies were also recorded for defect classification.^17^ All the treated teeth are associated with a non‐carious cervical lesion (NCCL):

- Seven teeth were classified as Class B+ (unidentifiable CEJ with the presence of a cervical step).
- Three teeth were classified as Class A+ (identifiable CEJ with the presence of a cervical step).
- One tooth was classified as Class B‒ (unidentifiable CEJ with no cervical step).

**TABLE 1:** Baseline clinical measurement and diagnosis of the five cases.

Case	Tooth (no.)	REC (mm)	PD (mm)	Recession type^15^	Phenotype	KTW (mm)	CEJ (A/B)^17^	Step (±)^17^
1	#19	4	1	RT1	Thick	2	B	+
	#20	3	2	RT1	Thick	4	B	+
	#21	4	1	RT1	Thick	3	B	+
	#22	4	1	RT1	Thick	3	B	+
2	#11	4	2	RT1	Thick	2	A	+
	#12	4	1	RT1	Thick	1	B	+
3	#19	3	2	RT1	Thick	2	B	+
	#20	3	1	RT1	Thick	2	A	+
	#21	4	1	RT1	Thick	2	A	+
4	#5	3	2	RT1	Thick	3	B	+
5	#3	4	2	RT1	Thick	2	B	–

Abbreviations: CEJ, cementoenamel junction; KTW, keratinized tissue width; PD, probing depth; REC, recession depth.

**TABLE 2:** Twelve‐month postoperative clinical measurements.

Case	Tooth (no.)	REC (mm)	PD (mm)	CAL (mm)	KTW (mm)	Phenotype
1	#19	2	2	4	3	Thick
	#20	1	2	3	5	Thick
	#21	1	2	3	4	Thick
	#22	0	2	2	4	Thick
2	#11	0	2	4	3	Thick
	#12	0	2	4	2	Thick
3	#19	2	1	3	2	Thick
	#20	0	2	2	3	Thick
	#21	0	2	2	4	Thick
4	#5	1	2	3	4	Thick
5	#3	1	2	3	3	Thick

Abbreviations: CAL, clinical attachment level; KTW, keratinized tissue width; PD, probing depth; REC, recession depth.

In cases where the anatomical CEJ was not clinically visible due to the presence of NCCL, the clinical CEJ was predetermined following the method described by Zucchelli et al.^18^ Specifically, the ideal vertical dimension of the interdental papilla was measured from the adjacent teeth and used as a reference to estimate the level of the clinical CEJ. The gingival margin position after surgery was expected to correspond to this predetermined clinical CEJ. Care was taken to differentiate cervical abrasion lines from the actual CEJ to avoid misinterpretation of the root coverage outcome.

The follow‐up period extended to 12 months. All patients provided informed consent for the surgical procedure, data collection, and publication of intraoral photographs.

### Recipient site preparation

**FIGURE 2:**
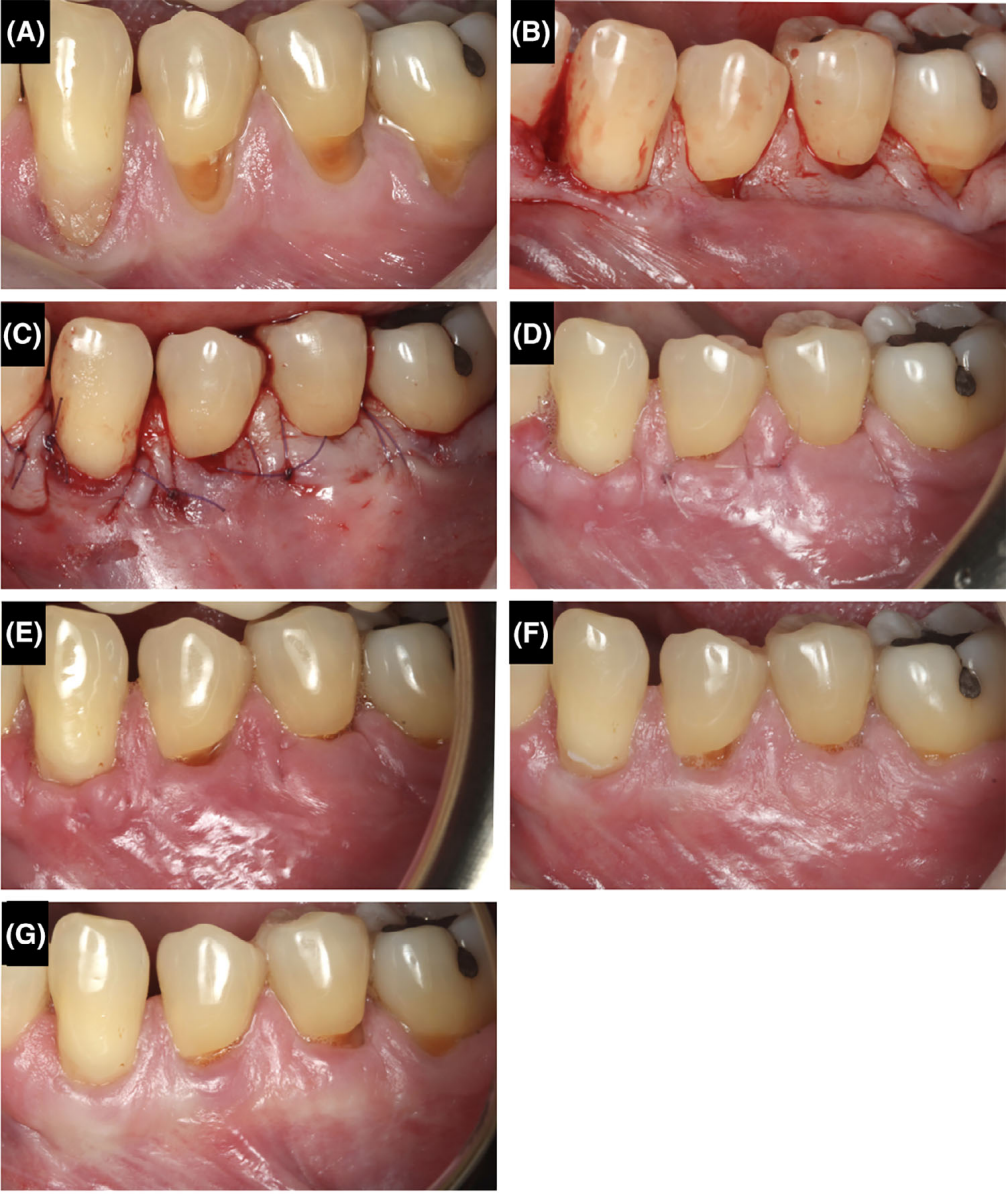
Case 1: (A) RT1 buccal gingival recession defects (3‒4 mm) were noted from the mandibular left first molar to the left canine. (B) Recipient site was tunneled full thickness with the use of a vestibular access incision (VAI). (C) After the microsurfaced acellular dermal matrix (mADM) was trimmed, inserted, and positioned, the mADM and the flap were secured dependently with sling sutures. (D) Two weeks postoperatively: uneventful healing was observed. (E) Four weeks postoperatively: excellent results were apparent. (F) Eight weeks postoperatively. (G) One year postoperatively: the gingival margin was stable. Gingival color and texture appear thicker and of a natural appearance.

**FIGURE 3:**
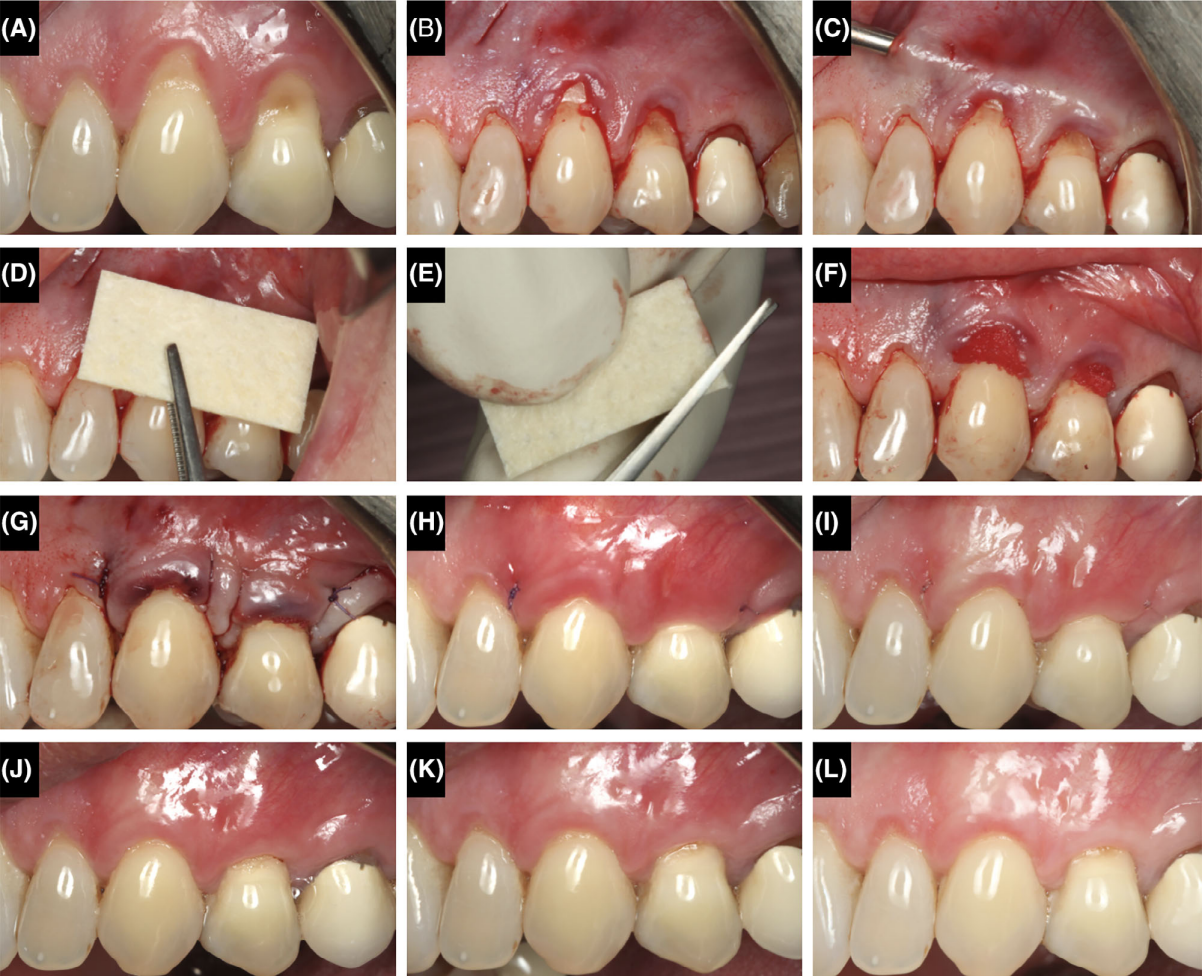
Case 2: (A) RT1 buccal gingival recession defects (4 mm) were observed from the maxillary left canine to the first premolar. (B) Recipient site was prepared using a vestibular access incision (VAI) placed in the alveolar mucosa mesial to tooth #23, along with sulcular incisions. (C) A full‐thickness tunnel was created to allow for complete coverage of the microsurfaced acellular dermal matrix (mADM) and adequate coronal advancement. (D) Appropriate dimensions of the mADM were measured. (E) mADM was trimmed dry to the correct size. (F) Final position of the mADM was confirmed. (G) mADM and the flap were secured using sling sutures. (H) Two weeks postoperatively: healing was uneventful, with gingival color appearing red and some swelling observed. (I) Four weeks postoperatively: the gingiva appeared pink and healthy. (J) Nine weeks postoperatively. (K) Four months postoperatively. (L) One year postoperatively: root coverage outcome remained stable.

**FIGURE 4:**
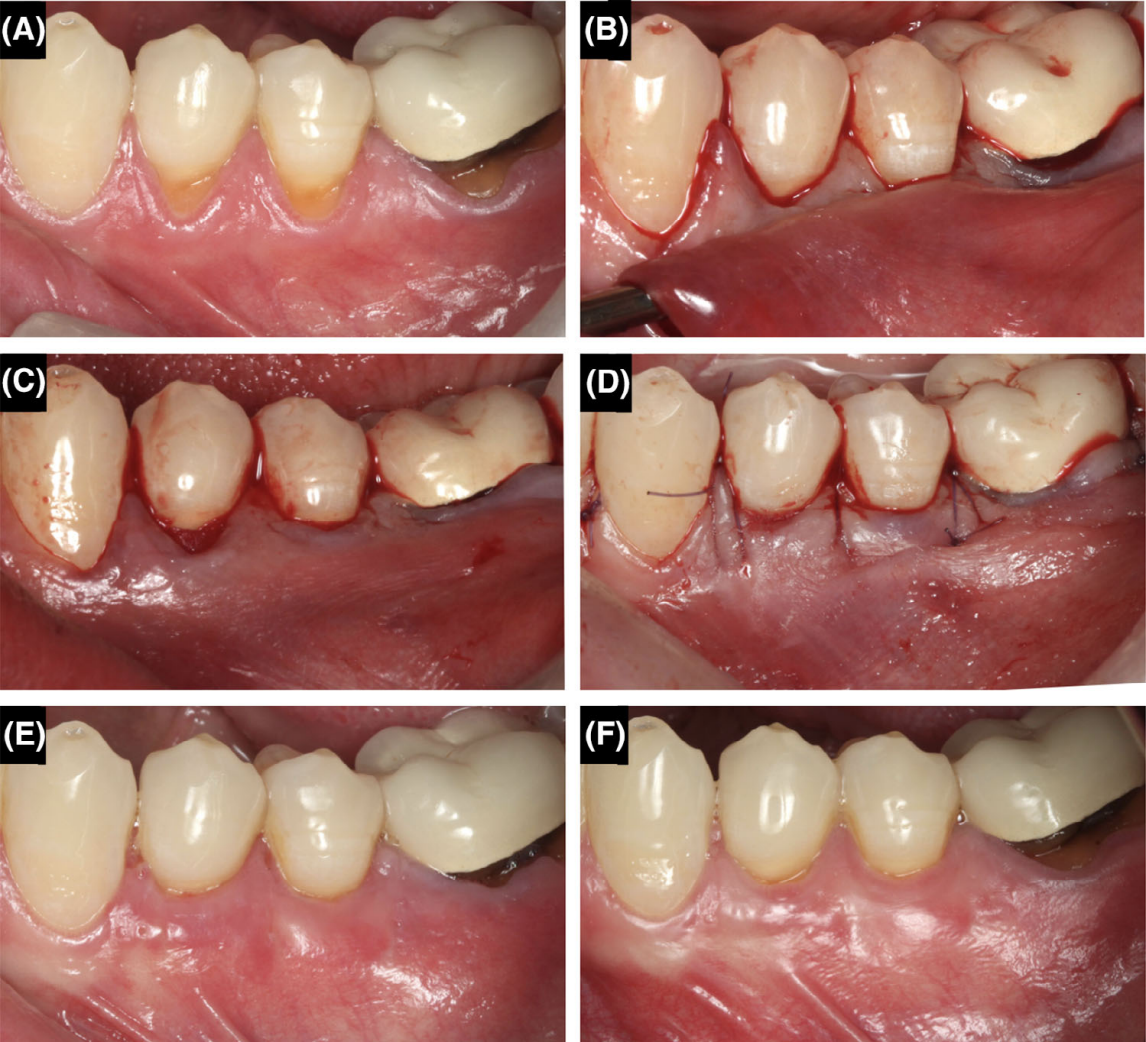
Case 3: (A) RT1 buccal gingival recession defects (3‒4 mm) were observed from the mandibular left first premolar to the first molar. (B) Tunnel flap was prepared with the full‐thickness approach using a vestibular access incision (VAI). (C) Microsurfaced acellular dermal matrix (mADM) was inserted and positioned without any folding. (D) mADM and the flap were sutured using sling sutures. (E) Four weeks postoperatively: healing was uneventful. Complete root coverage on the first and second premolar was observed. (F) One year postoperatively: root coverage outcome remained stable, with natural gingival color and texture.

**FIGURE 5:**
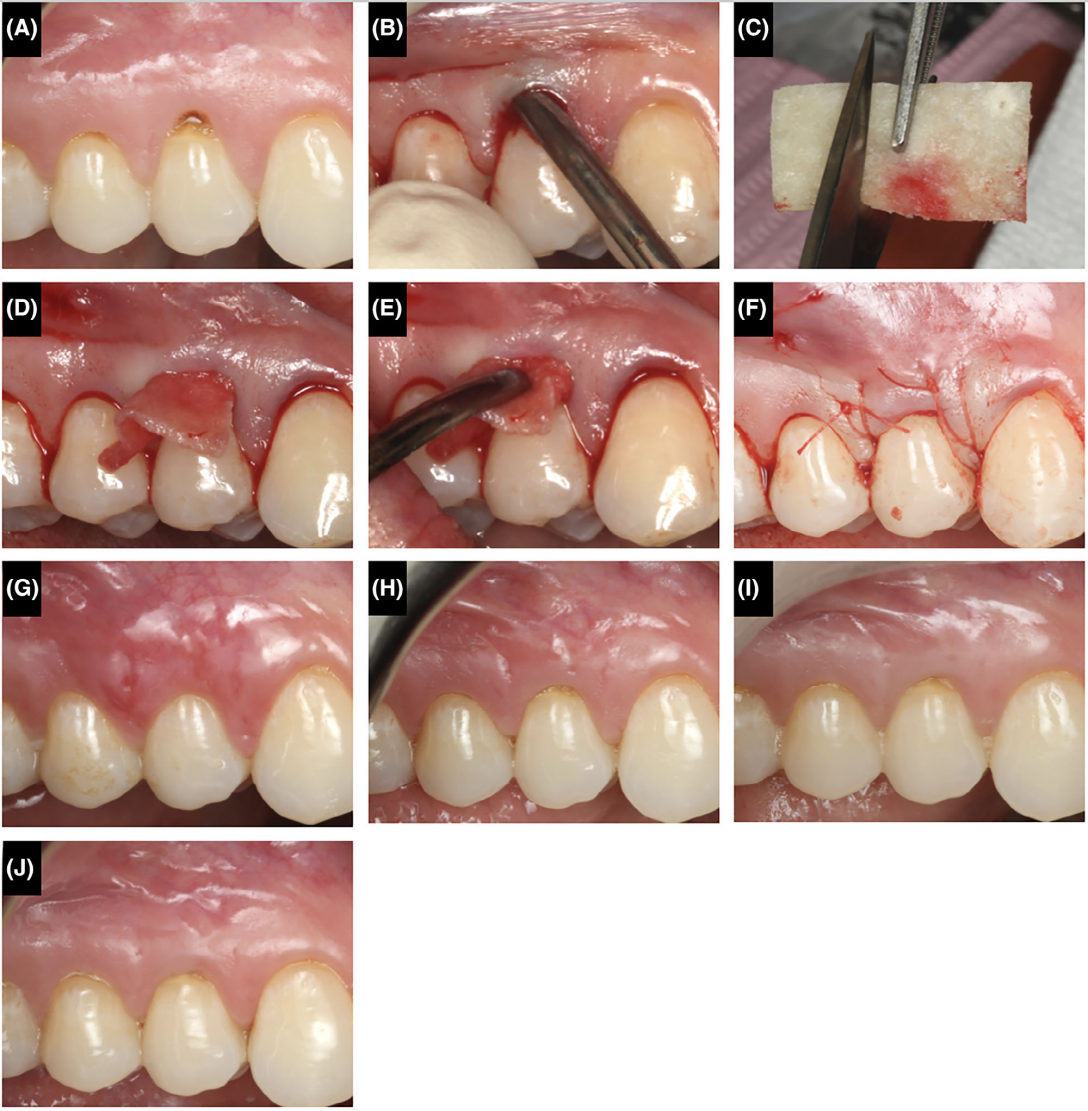
Case 4: (A) RT1 buccal gingival recession defect (3 mm) was observed at the maxillary right first premolar. (B) A pouch was prepared through the gingival sulcus of the tooth. (C) Microsurfaced acellular dermal matrix (mADM) was trimmed to a proper dimension. (D) mADM was inserted via the gingival sulcus. (E) mADM was placed into the prepared pouch. (F) mADM and the flap were secured with sling sutures. (G) Two weeks postoperatively: healing was uneventful, with gingival color appearing red and some swelling observed. (H) Three months postoperatively. (I) Five months postoperatively. (J) One year postoperatively: root coverage outcome remained stable, with natural gingival color and texture.

**FIGURE 6:**
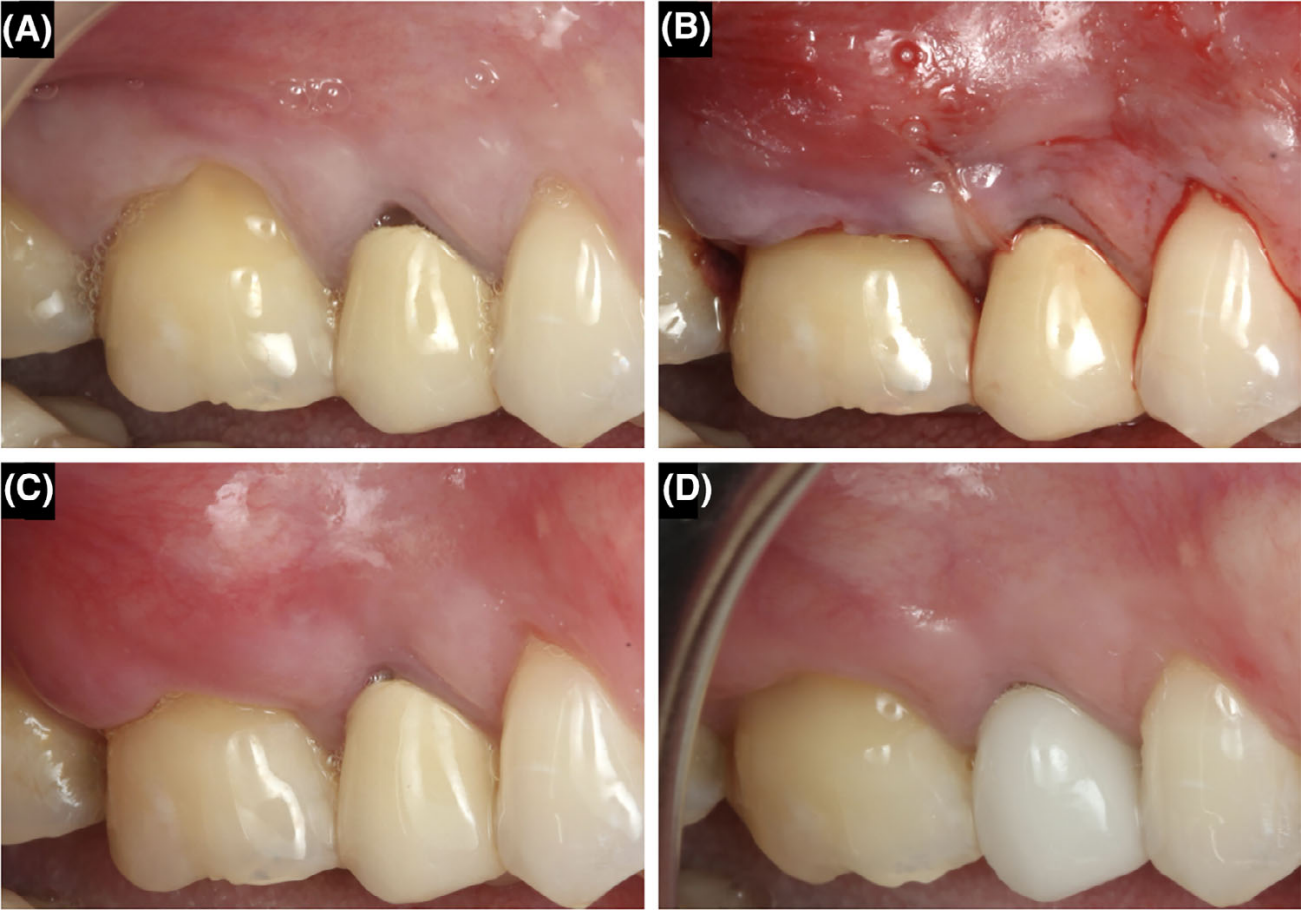
Case 5: (A) RT1 buccal gingival recession defect (4 mm) was observed at the maxillary right first molar. (B) Overlying flap and microsurfaced acellular dermal matrix (mADM) were sutured dependently using 5‐0 polyglycolic acid (PGA) sling suture. (C) Healing at 4 weeks demonstrates good increases in tissue thickness and root coverage with minimal inflammation. (D) One year postoperatively: the crown on tooth #4 was changed during the course of treatment. Root coverage and tissue thickness gains appear stable with natural soft tissue appearance.

After administering local anesthesia (2% lidocaine with 1:100,000 epinephrine; Lignospan, Septodont USA), the exposed root surfaces were instrumented using a universal ultrasonic insert and Gracey 7/8 curette. A 50 mg/mL tetracycline solution was then applied for 1 min to exposed root surfaces, followed by thorough irrigation with water. Odontoplasty was not performed on any cases. For multiple adjacent gingival recession defects (cases 1‒3) (Figures 2, 3, 4), a modified vestibular incision subperiosteal tunnel access^19^ was performed using a combination of sulcular incisions and a 5‒8 mm vestibular access incision (VAI). Initial tunnel preparation was carried out through the VAI apical to the mucogingival junction (MGJ), extending from the most anterior to the most posterior teeth planned for treatment. Once this was completed, full‐thickness tunnel preparation continued coronal to the MGJ until the gingival margin and base of the papilla were reached. To complete the recipient site, passive communication was established between the gingival margins of the target teeth and the vestibular tunnel apical to them. For single tooth gingival recession defects (cases 4 and 5) (Figures 5 and 6), a subperiosteal pouch technique was utilized to prepare the recipient site.^20^

### mADM preparation

In each case, the mADM was trimmed to the appropriate dimensions while still in its dehydrated form. Specifically, the width was standardized at 10 mm, and the length was determined by measuring from the mesial interdental area of the most mesially affected tooth to the distal interdental area of the most distally affected tooth, ensuring complete coverage of the recession sites. Due to its microsurfacing properties, mADM rapidly hydrates upon contact with liquid. To ensure thorough hydration with the patient's own blood, the mADM was inserted dry, directly into the prepared recipient site, without pre‐hydration with any other fluid.

### mADM insertion and suturing

For cases 1 and 2 (Figures 2 and 3), the mADM was introduced into the recipient site through the VAI and was passively delivered into final positioning.^19^ For case 3 (Figure 4), the mesial papilla of the most anterior tooth to be treated was elevated to create an entryway for the mADM at a more coronal position in order to avoid encroaching on the mental foramen. For cases 4 and 5 (Figures 5 and 6), mADM was inserted through the gingival margin into the subperiosteal pouch.^20^ Care was taken to distribute the material evenly in the recipient space, ensuring it did not fold or twist during insertion. Once properly adapted, the material was sutured together with the overlying flap using 5‐0 polyglycolic acid (PGA; PGA Resorba, Osteogenics Biomedical) or a copolymer of glycolic acid and caprolactone (PGA‐PCL; Glycolon, Osteogenics Biomedical) sutures via sling sutures. In all cases, each tooth being treated received its own sling suture. Finally, tissue adhesive (PeriAcryl 90HV, Glustitch, Delta) was applied over each suture knot to secure the knot, ensuring its stability and preventing displacement during the healing process.

### Postoperative instructions

Patients were instructed to avoid solid foods for 14 days and to use a 0.12% chlorhexidine solution (Colgate PerioGard Rinse, Colgate‐Palmolive) twice daily for the first 7 days. They were also prescribed systemic antibiotics for 7 days (amoxicillin 500 mg three times daily). For patients allergic to penicillin, azithromycin was prescribed instead (250 mg twice daily on the first day, followed by 250 mg once daily from days 2 to 5). For pain control, patients were advised to take 600 mg ibuprofen every 6 hours for the first 48 hours postoperatively. After the first week, patients were advised to use an extra‐soft toothbrush and to clean only the supragingival areas. Normal brushing resumed 4 weeks postoperatively. Sutures were left to be resorbed naturally and were not removed during the 2‐ or 4‐week postoperative visits.

### Statistical analysis

Descriptive statistics were used to present the clinical outcomes, with means ± standard deviations. Pair *t*‐tests were used to statistically compare the clinical outcomes between baseline and 12 months. A *p*‐value threshold of 0.05 was set for statistically significance. The analyses were performed at the Ohio State University (Columbus, Ohio, USA).

## RESULTS

### Demographic data

A total of five patients (three females and two males) with RT1 gingival recession were included in this case series. The mean age of the patients was 50 years, ranging from 38 to 62 years. A total of 11 teeth were treated, with individual cases involving between one and four teeth. Two patients had a single‐tooth recession defect, while the remaining three had multiple adjacent recession defects.

### Clinical outcomes

The clinical measurements at the 12‐month follow‐up are shown in Table 2. The clinical outcome comparison between baseline and 12‐month follow‐up is summarized in Table 3. At the 12‐month follow‐up, significant improvements were observed in REC, CAL, and KTW. The mean REC decreased from 3.64 ± 0.50 mm at baseline to 0.73 ± 0.79 mm at 12 months (*p* = 0.00000327), demonstrating a substantial gain in root coverage. Similarly, KTW increased from 2.32 ± 0.81 mm to 3.36 ± 0.92 mm (*p* = 0.0000227), indicating a significant augmentation in keratinized tissue. The CAL showed a statistically significant improvement, decreasing from 5.00 ± 0.63 mm at baseline to 3.00 ± 0.77 mm at 12 months (*p* = 0.00000646). In contrast, probing depth (PD) increased slightly from 1.45 ± 0.52 mm to 1.91 ± 0.30 mm, but this change was not statistically significant (*p* = 0.0531).

**TABLE 3:** Clinical outcomes at 12 months.

Outcome (mean ± SD)	Baseline	12 months	Baseline‒12 months	*p*‐value
REC (mm)	3.64 ± 0.50	0.73 ± 0.79	2.91 ± 1.04	0.00000327^*^
PD (mm)	1.45 ± 0.52	1.91 ± 0.30	0.45 ± 0.69	0.0531
KTW (mm)	2.32 ± 0.81	3.36 ± 0.92	1.00 ± 0.45	0.0000227^*^
CAL (mm)	5.00 ± 0.63	3.00 ± 0.77	2.00 ± 0.77	0.00000646^*^
CRC (*n*/%)		5/45.5		
RC (%)		80		
Phenotype	Thick	Thick		

Abbreviations: CAL, clinical attachment level; CRC (*n*/%), complete root coverage (number/percentage); KTW, keratinized tissue width; PD, probing depth; RC (%), root coverage percentage; REC, recession depth; SD, standard deviation.

* *p*‐Value < 0.05, statistically significant difference.

The mean percentage of root coverage (%RC) at 12 months was 80.0%. The mean recession reduction was 2.91 ± 1.04 mm.

Complete root coverage (CRC) was achieved in five sites (45.5%), specifically in:

- Case 1: tooth #22
- Case 2: teeth #11 and #12
- Case 3: teeth #20 and #21

The gingival phenotype remained thick in all cases before and after the procedure.

No adverse events were observed at any postoperative visits for all patients. At the 2‐ and 4‐week follow‐ups, only minimal inflammation was noted and all patients reported minimal postoperative discomfort. Importantly, there were also no signs of graft necrosis at any postoperative visit. Patients consistently reported a positive recovery experience, with none reporting any unusual odors or tastes associated with necrotic graft tissue. By the 12‐month postoperative follow‐up, all treated sites demonstrated increased root coverage, as shown in Tables 2 and 3. Additionally, in case 5, while tooth #3 was the primary site treated for gingival recession, the crown on tooth #4 was replaced during the course of treatment. Importantly, all patients reported complete resolution of tooth sensitivity and expressed satisfaction with the final esthetic outcome, effectively addressing their primary concerns regarding periodontal health and sensitivity associated with gingival recession.

## DISCUSSION

Over the last 25 years, there have been minimal modifications to the physical properties of ADM. Although ADM has demonstrated clinical success, shortcomings remain when compared to autogenous tissue in the treatment of mucogingival deformities.^5^, ^6^ This case series introduces a novel, modified ADM that employs microcuts to increase the material's surface area—a design rationale intended to potentially facilitate graft integration and improve clinical outcomes.^13^ In a previous study, Marinelli et al.^13^ demonstrated that microsurfaced grafts enhance integration into the wound bed and promote effective healing by increasing the surface area at the graft‐to‐host interface, leading to greater cellular infiltration and graft thickness. Additionally, the material can be inserted without pre‐hydration and rapidly absorbs blood. In comparison, other soft tissue allografts need either a pre‐hydration process for several minutes^5^ or are packaged in a glycerol saline solution.^21^

In this case series, five patients (mean age 50 years) with a total of 11 RT1 recession defects were treated using mADM. At the 12‐month follow‐up, significant improvements were observed: the mean REC decreased markedly from 3.64 ± 0.50 mm to 0.73 ± 0.79 mm, and the KTW increased from 2.32 ± 0.81 mm to 3.36 ± 0.92 mm. Additionally, the CAL improved significantly, decreasing from 5.00 ± 0.63 mm to 3.00 ± 0.77 mm. The overall mean %RC reached 80.0%, with CRC achieved in 45.5% of the treated sites. Importantly, all patients reported complete resolution of tooth sensitivity, minimal postoperative discomfort, and no adverse events were observed, supporting the potential of mADM as a viable treatment option for gingival recession defects. Furthermore, in all cases presented, root coverage was increased with good stability up to the 12 months.

A noteworthy finding in this case series was the complete resolution of tooth sensitivity in all patients at the 12‐month postoperative follow‐up. A systematic review by Antezack et al. highlighted that dentin hypersensitivity suppression is significantly associated with the %RC achieved and the reduction of exposed root dentin,^22^ underscoring the critical role of CRC in addressing tooth sensitivity. The complete resolution of dentin hypersensitivity observed in this case series could be attributed to the high root coverage percentage (80%), similar to the mean root coverage of 80.9%^23^ of Miller I and II^24^ reported by a systematic review from AAP. These outcomes may indicate that mADM could provide predictable root coverage and improved the dentin hypersensitivity.

Despite the promising outcomes observed in this case series, certain limitations must be acknowledged. First, the lack of a clearly defined clinical CEJ as a reference point for evaluating outcomes was a challenge due to unaddressed NCCL. A major challenge in root coverage procedures is accurately identifying the CEJ, particularly when it is obscured by a NCCL. In this study, we utilized the clinical CEJ predetermination method described by Zucchelli et al.^18^ to estimate the soft tissue margin after healing. This approach relies on the measurement of ideal papilla height as a reference point, allowing for a predictable assessment of root coverage outcomes. However, the methods to determine the level of the lost CEJ are relatively subjective; therefore, the final result may be compromised in cases where the estimated CEJ is placed more coronally than the previously destroyed CEJ. Second, residual cervical abrasion lesions were not treated in all cases, which may have influenced the final outcomes. Surgical root coverage procedures are less likely to achieve full coverage^25^, ^26^ and may have an increased risk of gingival recession recurrence^27^ when performed at sites where gingival recession is associated with NCCL. However, studies by Santamaria et al.^26^, ^28^ showed treating recession defects with NCCL using restoration materials with SCTG does not alter the root coverage outcome. Notably, the use of resin composite with SCTG resulted in improved gingival contour and greater resolution of dentin hypersensitivity.^26^ Third, patient‐reported outcome measures were not available due to the retrospective nature of this case series. As a result, we were unable to provide quantitative records assessing esthetic outcomes and dentin hypersensitivity. Fourth, all surgical procedures and clinical assessments were performed by the same examiner, who was not blinded. This introduces a potential bias that may influence the objectivity of the clinical measurements. Fifth, due to the retrospective nature of the study, examiner calibration was not performed, which may have affected the consistency and reliability of clinical assessments. Sixth, the recipient site preparations across the five cases were not standardized, introducing potential variability in the results. Last, the small sample size limits the generalizability of these findings.

However, the treatment resulted in excellent root coverage without complications such as graft exposure or infection. Additionally, it is worth noting that all patients reported minimal postoperative discomfort and were satisfied with the esthetic outcome. Nonetheless, future randomized clinical trials research with larger sample sizes, standardized protocols, and longer follow‐up periods will be essential to compare the initial healing and long‐term outcomes of this innovative matrix with other treatment modalities for recession defects.

## CONCLUSION

Within the limits of this retrospective case series, our findings indicate that mADM may offer a viable treatment alternative for RT1 gingival recession defects for 12‐month period. Future randomized clinical trials are necessary to compare mADM with other options to treat recession defects.

## AUTHOR CONTRIBUTIONS

Yu‐Chang Wu contributed to data analysis, data interpretation, manuscript preparation, and final approval of the manuscript. Guo‐Liang Cheng contributed to data interpretation, manuscript preparation, and final approval of the manuscript. Shaun Rotenberg contributed to the concept of the work, data collection, data interpretation, manuscript preparation, and final approval of the manuscript. Figure 1a,b was provided by Osteogenics Biomedical.

## CONFLICT OF INTEREST STATEMENT

This case series did not receive any grant from any funding agency in the public, commercial, or not‐for‐profit sectors. Dr. Shaun Rotenberg has previously received consulting and lecture fees from Osteogenics Biomedical. However, the surgeries included in this study were performed prior to any consulting he did on soft tissue for Osteogenics. Furthermore, he did not receive any financial compensation related to the cases presented in this study. The authors declare no other conflicts of interest and no financial interests in the companies whose materials were included in this article.

## PATIENT CONSENT STATEMENT

The authors received verbal and written consent for treatment from all five patients.

## Data Availability

The data supporting the findings of this study are included in this article.
